# The construct validity of an abridged version of the general self-efficacy scale for adults with attention-deficit/hyperactivity disorder

**DOI:** 10.3389/fpsyt.2023.1212961

**Published:** 2023-11-06

**Authors:** Tatiana Skliarova, Henrik Pedersen, Hege Hafstad, Jonas Rennemo Vaag, Mariela Loreto Lara-Cabrera, Audun Havnen

**Affiliations:** ^1^Department of Mental Health, Faculty of Medicine and Health Sciences, Norwegian University of Science and Technology (NTNU), Trondheim, Norway; ^2^Nidaros Community Mental Health Center, Division of Psychiatry, St. Olavs University Hospital, Trondheim, Norway; ^3^Vårres Regional User-Led Center Mid-Norway, Trondheim, Norway; ^4^Department of Psychology, Inland University of Applied Sciences, Lillehammer, Norway; ^5^Nidelv Community Mental Health Center, Department of Mental Healthcare, St. Olavs Hospital, Trondheim University Hospital, Trondheim, Norway; ^6^Department of Psychology, Norwegian University of Science and Technology (NTNU), Trondheim, Norway

**Keywords:** attention deficit disorder with hyperactivity (ADHD), self-efficacy, mental disorders, reliability, validation, psychometrics, adults

## Abstract

**Objectives:**

The General Self-Efficacy (GSE) scale is a validated self-rated questionnaire increasingly used in mental health research. However, despite several psychometric advantages of the GSE scale, its validity in those diagnosed with attention-deficit/hyperactivity disorder (ADHD) has not yet been examined. Moreover, a shorter version of the GSE scale would contribute to a more rational use of resources in extensive multivariate studies. Therefore, as self-rated scales to measure self-efficacy in this population are lacking, the current study aims to develop a condensed version of the GSE for adults with ADHD.

**Methods:**

A group of patient collaborators (user representatives) from an ADHD organization and health professionals shortened the original 10-item GSE scale to six items and evaluated the content validity of the revised scale. Second, 525 potential participants were invited to participate in a cross-sectional study conducted in 2021 (between January 19th and February 7th). Of them, 403 filled out the GSE-6 for ADHD and two scales measuring psychological well-being and mental health (the five-item World Health Organization Well-Being Index, WHO-5, and the four-item Patient Health Questionnaire, PHQ-4). The psychometric properties of the new scale were examined, testing *a priori* formulated hypotheses.

**Results:**

The brief GSE-6 for ADHD displayed good internal consistency with a Cronbach’s α of 0.907. No floor or ceiling effect was detected. Exploratory and confirmatory factor analyses supported a one-factor structure. The GSE-6 also showed a moderate positive correlation with the WHO-5 (*r_s_* = 0.578) and a moderate negative correlation with the depression and anxiety rating scale PHQ-4 (*r_s_* = −0.595).

**Conclusion:**

The 6-item GSE for ADHD was evaluated to have good content validity. The scale demonstrated good psychometric properties. The results indicate that the GSE-6 may help assess self-efficacy in adults with ADHD.

## 1. Introduction

Attention-deficit/hyperactivity disorder (ADHD) is a neurodevelopment condition characterized by inattention, impulsivity and hyperactivity (1). In addition, recent studies have shown that the prevalence of this disease among children is, on average 2.2%, with a wide range of the country’s income level from 0.1 to 8.1% (2). At the same time, the prevalence of ADHD among adults was even higher and amounted to 2.8% (the prevalence also depended on the country’s income level), and 57% of the adults surveyed had a history of ADHD in childhood (2). However, despite these indicators, adult ADHD in Europe is still underdiagnosed and undertreated (3, 4). Moreover, ADHD is associated with psychosocial impairment, including a lower likelihood of finishing higher education, and work-related difficulties (5, 6). People with ADHD have a higher risk of accidents and drug and alcohol abuse (7, 8). Further, ADHD has a high comorbidity with other psychiatric conditions, such as anxiety and depression (9). Evidence indicates that those with ADHD also have lower psychological protective factors, such as self-efficacy (10, 11).

Self-efficacy is understood as a person’s belief in the ability to control the complex demands of the environment through adaptive actions (12). Self-efficacy may be conceptualized as a protective factor when facing stressors. Studies have shown that self-efficacy strongly predicts self-management abilities, such as coping behaviors, performance, and perseverance in complex challenges (13, 14). Furthermore, higher self-efficacy may be associated with less psychological distress following daily stress (15). Therefore, an individual’s perceived self-efficacy may be critical for how well they cope with psychiatric symptoms or mental disorders. In support of this, self-efficacy has also been found to predict better physical health outcomes (16) and mediate the association between stressful life events and depression (17). Higher self-efficacy in those with chronic diseases has also been found to reduce the risk of depression, and a longitudinal study revealed that those with high self-efficacy were less likely to have had a major depressive disorder over the 6-year study period (18).

For adults with ADHD, higher self-efficacy is also associated with lower parenting stress (19). Self-efficacy may also be vital for individuals with ADHD seeking treatment or other mental health care. For individuals with ADHD, increased self-efficacy may lead to an increased belief that one can deal with everyday challenges frequently experienced by adults with ADHD through one’s actions (10). Self-efficacy has a critical role in changing lifestyle, such as adopting a new behavior, maintaining motivation and reinforcing new behavior, including overcoming possible failures (12). For clinicians, these are all critical processes in clinical work with patients with ADHD (11).

Self-report questionnaires with good psychometric abilities are needed to measure self-efficacy. According to Mokkink et al. (20), psychometric assessment of instruments is critical, as it affects the results that determine treatment tactics, and the use of invalid instruments can lead to distorted results (21). The General Self-Efficacy (GSE) questionnaire was initially developed by Jerusalem and Schwarzer in 1979 as a self-assessment scale with 20 items. Later the scale was reduced to 10 items (GSE-10) (22). The GSE-10 has been translated into several languages and displays good psychometric properties (23). The GSE-10 has been validated in mental health settings among psychiatric outpatients in Spain (24) and individuals with schizophrenia in China (25). In addition, the GSE scale has been demonstrated as a positive predictor of improved mental health (26) and a mediator between self-management (health literacy) and healthy habits (27).

The GSE-10 has been used to measure self-efficacy in mental health settings (24, 25) and in adults with ADHD (28, 29). However, due to the attention difficulties experienced by those with ADHD, short scales with as few items as possible are preferable in clinical contexts. Moreover, in research settings, response burden is frequently mentioned as a concern when conducting studies, suggesting the pragmatic need to reduce the number of items (30). Item reduction is also crucial because participants must often complete multiple self-report measures to save time and reduce their burden (30, 31).

Previous studies have reduced the number of items of the GSE. Romppel et al. (31) developed a six-item version of the GSE scale validated in a nonclinical sample and a sample of patients at risk for heart failure. The results of the research demonstrated good internal consistency of the scale (Cronbach’s alpha was between 0.79 and 0.88), good test–retest reliability (*r* = 0.50 and 0.60 after 12 and 28 months, respectively), a positive correlation of the scale with social support and mental health, and a negative correlation with symptoms of depression and anxiety. Bonsaksen et al. (32) developed a seven-item version of the GSE scale and tested its validity on adult Norwegians suffering from morbid obesity. These validation studies demonstrated adequate psychometric properties (31, 32), which support that the GSE is suitable as a brief scale. Even though a short version of the GSE could serve as a valuable, brief, and easily administered self-report scale to measure self-efficacy in adults with ADHD, studies which include the patients’ view of burden concerning the answering the GSE are lacking. In addition, there are no studies to support whether adults with ADHD consider the GSE-scale valuable.

However, in order to adapt a brief version of the GSE-10 tailored to individuals with ADHD, we used an expert panel of adults with ADHD and health professionals to guide the selection of appropriate items. The first aim of this study is to develop and validate a short six-item version of the GSE by involving adults with ADHD and user representatives from the Norwegian user-led ADHD organization. The second aim is to examine the construct validity and scale internal consistency of the revised GSE-6 questionnaire in a sample of adults diagnosed with ADHD.

## 2. Methods

### 2.1. Study 1: Development of an abridged version of general self-efficacy-6 for attention-deficit/hyperactivity disorder

When planning and conducting this validation study, we followed the methodology proposed by the Consensus-based Standards for the Selection of Health Measurements Instruments, COSMIN (20), and the Strengthening the Reporting of Observational Studies in Epidemiology, STROBE (33). The development of the condensed version of the GSE-6 for the ADHD scale followed two phases.

#### 2.1.1. Phase 1

In the first phase, we reduced the number of items from 10 to six. This stage was conducted in collaboration with five health professionals (two nurses, one psychiatrist and two psychologists) and user representatives from Norwegian ADHD organization – Vårres Regional User-led Center Mid-Norway. The reduction of items was based on consensus reached through group discussions by the health professionals and the user representatives (34). The role of user representatives was to explore the content validity of the items on the brief scale, review their relevance, and provide feedback about the scale’s language, ease of use, and interpretability.

#### 2.1.2. Phase 2

In the second phase, adults diagnosed with ADHD evaluated the experience of answering the GSE-6 by completing the QQ-10. In this phase, 18 adults from the Norwegian ADHD user-led organization were invited to participate, and 16 participants completed the questionnaires.

#### 2.1.3. Participants, procedures, and measures

The 16 recruited adults completed a paper version of the GSE-6 scale and QQ-10. On average, the testing group took 1 to 2 min to complete the GSE-6 scale. Data collection did not include names or other direct identifiers to ensure anonymity and confidentiality. Data were stored as an anonymous SPSS file. The SPSS file was accessible to authorized researchers and was protected with a two-factor authentication login system.

#### 2.1.4. QQ-10

The QQ-10 is a10-item questionnaire designed to assess the opinion of patients about their experience using questionnaires during medical care. It includes a five-point Likert scale relating to the subject’s agreement with statements about their experience using the questionnaire (35). In the present study, two responses are produced with this tool: positive value (communication, relevance, ease of use, comprehensiveness, pleasantness and willingness to repeat) and negative burden (excessively long, too simple questions, complicated, and upsetting). The score ranges from 0 to 4 for both domains. Then, raw scores are converted on a scale from 0 to 100 (where 0 is defined as the worst value, and 100 is defined as the best possible representation of the questionnaire) (36). In this research, Cronbach’s α = 0.866 for the “value” domain and 0.760 for the “burden” domain.

#### 2.1.5. Statistical analysis

The SPSS (SPSS v. 28, IBM Corp., Armonk, NY, United States) was used for statistical analysis. Mplus version 8.8 (37) was used to conduct exploratory factor analysis (EFA) and confirmatory factor analysis (CFA). The results of the QQ-10 were evaluated using descriptive statistics, including the mean, frequency, standard deviation (*SD*), and percentage.

### 2.2. Study 2: Validation of the general self-efficacy-6

The construct validity, reliability, and floor or ceiling effects were investigated using predefined hypotheses (Table 1). We assessed the internal consistency for the GSE-6 for patients with ADHD using Cronbach’s α and evaluated the floor or ceiling effects. We evaluated the correlation between self-efficacy, well-being, and self-reported depression using Spearman’s *r_s_* between GSE-6, the five-item well-being scale (WHO-5), and the four-item Patient Health Questionnaire (PHQ-4). Moreover, we assessed structural validity using EFA and CFA. The procedure for validating the GSE-6 for ADHD was based on an earlier validation study of the WHO-5 (38).

**Table 1:** Hypotheses testing and results.

Hypotheses	Results	Decision
Internal consistency: Cronbach’s α > 0.7 for GSE-6	Cronbach’s α = 0.907 [95% CI 0.892–0.920]	Accepted
No floor or ceiling effect (less than 15% of patients have extreme scores)	0.5% of cases obtained the minimum score; 6.5% of cases obtained the maximum	Accepted
GSE-6 for ADHD has a unidimensional structure	One-factor structure, eigenvalue = 4.624, RMSEA = 0.101 [90% CI 0.073–0.131], CFI = 0.994, TLI = 0.991, SRMR = 0.030	Accepted
Positive correlation between GSE-6 for ADHD and WHO-5	Spearman’s *r_s_* = 0.578	Accepted
Negative correlation between GSE-6 for ADHD and PHQ-4	Spearman’s *r_s_* = −0.595	Accepted

#### 2.2.1. Hypothesis testing

We defined the following *a priori* hypotheses based on previous studies:

1. The internal consistency for GSE-6 is more than acceptable: We expected a Cronbach’s α > 0.7 for GSE-6 (39);
2. Floor or ceiling effect: We expected no floor or ceiling effect (less than 15% of patients have extreme scores) (40);
3. Factor structure: We expected the GSE-6 to have a one factor structure (24);
4. Correlation with well-being: We expected the GSE-6 to be positively correlated (Spearman’s *r_s_*) with the WHO-5 scale (41);
5. Correlated with mental health problems: We expected the GSE-6 to be negatively correlated (Spearman’s *r_s_*) with the PHQ-4 scale (42).

#### 2.2.2. Participants, procedures, survey elements and measures

We recruited Norwegian-speaking adults by sending an email invitation to 525 potential participants registered in the Norwegian ADHD user-led organization. Participants were asked to send the e-mail invitation and the web link to other possible participants, and the link was also shared via social media (Vårres Regional User-led Center Mid-Norway). On the first page, participants read an online consent form, providing information about data storage policies and outlining the study purpose, survey length, and data use. By clicking “I agree,” the participants confirmed that they had read the information about the validation study and that they agreed to participate. A total of 403 adults consented to participate, and data from these were used in further analyses.

Several precautions were taken to ensure anonymity and confidentiality. No identification list was created, and data collection did not include names, IP addresses, or other direct identifiers. In addition, to avoid multiple responses from the same individual, the survey settings were set to refuse responses from the same IP addresses. Data were stored as an anonymous SPSS file. The SPSS file was protected with a two-factor authentication login system.

#### 2.2.3. Survey elements and measures

The self-rated survey took 15 min to complete using Questback software. Data were collected from January 19 to February 7, 2021.

#### 2.2.4. Data collection and measures

Participants reported demographic data, including gender, educational level, age, marital and work status.

##### 2.2.4.1. General self-efficacy-6 for attention/deficit-hyperactivity disorder (GSE-6 for ADHD)

The GSE-6 items for ADHD (Section 3.1 provides an overview of the items) were ranked in the same way as the GSE-10 using a four-point scale from 1 (“not at all true”) to 4 (“exactly true”). The total score ranged from six to 24, where the minimum score equals the lowest level of general self-efficacy, and the higher score equals the highest level.

##### 2.2.4.2. The five-item world health organization 5-item well-being index (WHO-5)

The WHO-5 is a reliable self-assessment tool comprising five items that evaluate different dimensions of well-being. Participants respond to statements such as “I have felt cheerful and in good spirits,” “I have felt calm and relaxed,” and “I have felt active and vigorous” using a scale ranging from 0 (indicating “at no time”) to 5 (indicating “all the time”) (38). The scale’s scoring ranges from zero, representing the lowest level of perceived well-being, to 25, reflecting a higher perception of well-being. The validity of the WHO-5 has been previously confirmed through validation with a Norwegian sample (38). Its Cronbach’s α is 0.868 in our study.

Well-being encompasses a spectrum of emotional aspects that can significantly impact an individual’s self-efficacy beliefs. Recent studies have highlighted a notable link between self-efficacy and subjective well-being (40, 41, 43). Furthermore, it has been reported that high levels of well-being are associated with increased self-efficacy (44). When establishing the convergent validity of the self-efficacy scale, our working hypothesis was that self-efficacy would demonstrate a positive correlation with well-being.

##### 2.2.4.3. Patient health questionnaire for depression and anxiety (PHQ-4)

PHQ-4 (45) is an ultra-brief instrument comprising four items that assess self-reported symptoms of depression and anxiety. Specifically, two of the items focus on depressive symptoms (“Over the last 2 weeks, how often have you been bothered by the following problems?”: ‘Feeling down, depressed, or hopeless’ and ‘Little interest or pleasure in doing things’), while the other two items pertain to anxiety symptoms (“Over the last 2 weeks, how often have you been bothered by the following problems?”: ‘Feeling nervous or anxious or on edge’ and ‘Not being able to stop or control worrying’). Participants provide responses on a 0–3 Likert-type scale, where zero corresponds to “not at all” and three corresponds to “nearly every day.” A higher total score indicates more severe symptomatology. The PHQ-4’s validation has been previously demonstrated using a Norwegian sample (45, 46). Its Cronbach’s α is 0.865 in the present study.

Both self-efficacy and anxiety are rooted in an individual’s beliefs regarding their health and capabilities. Research has indicated a relationship between self-efficacy and mental health issues (31, 40, 42, 47). Therefore, when establishing convergent validity, our underlying hypothesis was that self-efficacy would exhibit a negative correlation with mental health problems.

#### 2.2.5. Statistical analysis

Data cleaning and initial statistical analysis for Study 2 were conducted using SPSS (SPSS v. 28, IBM Corp., Armonk, NY, United States). The data contained no missing values. Descriptive statistics include the mean, frequency, SD, and percentages. We also calculated floor or ceiling effects, and this was implied if more than 15% of participants obtained the highest or lowest score, respectively (48). Spearman’s rho was used for correlations between GSE-6 and other measures. To assess internal consistency, we used Cronbach’s alpha. A value more than 0.7 has been suggested to indicate satisfactory internal consistency (20, 49).

An exploratory factor analysis (EFA) with Oblique Geomin rotation was conducted to assess the factor structure of the GSE-6. Criteria for conducting the EFA were: a sufficiently large sample size, which includes at least 400 participants for conducting EFA (50, 51), a correlation matrix with at least some correlation coefficients at or above r ≥ 0.3, a significant Bartlett’s test of sphericity (*p* < 0.05), a Kaiser–Meyer–Olkin ≥ 0.6, and normally distributed data without outliers (52).

Given the six-items, and that it is recommended that factors have three indicators each (52), the EFA was predefined to compare a one-factor and a two-factor solution. The decision on the number of factors to extract was based on several criteria: The Kaiser criterion of eigenvalues >1.0, inspection of scree plot, parallel analysis, and a theoretical consideration of the content of the indicators. In addition, several fit indices were applied to indicate model fit: Standardized Root Mean Square Residual; SRMR (53) values less than 0.8, Root Mean Square Error of Approximation; RMSEA (54) values below 0.05 to indicate close fit, values between 0.05 and 0.08 to indicate fair fit and values between 0.08 and 0.10 (with the upper 95% confidence interval equal to or below 0.10) to indicate poor fit. The Comparative Fit Index (CFI) should be greater than 0.90 and non-Normed Fit index (Tucker-Lewis index; TLI) greater than 0.95 (54) to indicate good fit. The CFA model was defined as a one-factor solution without correlated error terms, using the same fit indices as for the EFA. To allow for test of measurement invariance between gender, seven participants who did not report their gender as woman or man were excluded from the analysis, thus n = 396 were included in the CFA. Measurement invariance was tested in a stepwise manner. Configural invariance was supported if the number of factors and indicator-factor patterns were equal across groups. For metric invariance factor loadings were constrained equal across groups and for scalar invariance the factor loadings and thresholds were constrained equal. Nested models were compared with the Mplus DIFFTEST option (37). In addition, models were evaluated in terms of change (Δ) in fit indices, with ΔCFI ≥ − 0.01 and ΔRMSEA <0.015 as threshold values, as recommended by Chen (55). The Weighted Least Squares Means and Variance adjusted (WLSMV) estimator was used for both EFA and CFA, due to the ordinal Likert scale of the GSE-6.

## 3. Results

### 3.1. Results for study 1

#### 3.1.1. General self-efficacy-6 for attention-deficit/hyperactivity disorder

The following items from the GSE-10 were retained in the revised GSE-6 for adults:

Item 1: “I am confident that I could deal efficiently with unexpected events” (GSE-10 Item 4).

Item 2: “Thanks to my resourcefulness, I know how to handle unforeseen situations” (GSE-10 Item 5).

Item 3: “I can solve most problems if I invest the necessary effort” (GSE-10 Item 6).

Item 4: “I can remain calm when facing difficulties because I can rely on my coping abilities” (GSE-10 Item 7).

Item 5: “When I am confronted with a problem, I can usually find several solutions” (GSE-10 Item 8).

Item 6: “I can usually handle whatever comes my way” (GSE-10 Item 10).

#### 3.1.2. QQ-10 results

**Figure 1:**
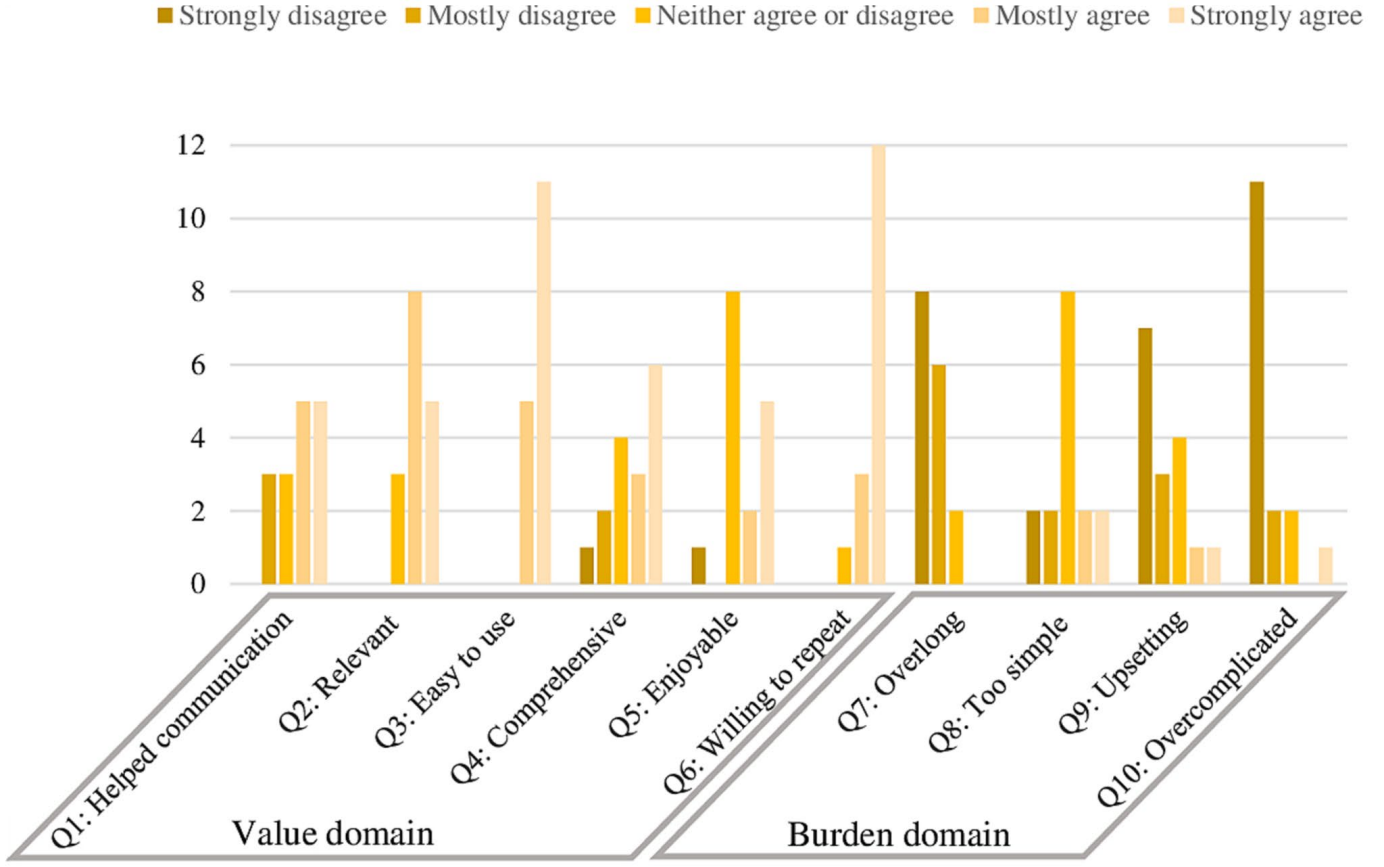
Items’ distribution QQ-10.

The QQ-10 results for the GSE-6 for ADHD scale revealed that the mean was 77% (*SD* = 18.3) for the “positive value” domain, and 18% (*SD* = 13.8) for the domain “negative burden.” The mean for each individual item assessing positive value was more than 2 (range 2.63 to 3.69 – raw values), that is, the participants primarily answered, “Mostly agree” and “Strongly agree” to questions from the value domain. For the negative burden domain, the mean was less than or equal to 2 (0.63 to 2.00), which means that the participants primarily chose the response options “Mostly disagree” and “Strongly disagree” when answering the burden domain questions. In the second burden domain, the only item that received a mean of 2.00 was Item 8, reflecting that the questions in the GSE-6 for ADHD were “too simple” for participants. The distribution of QQ-10 responses for the positive value domain and the negative burden domain is presented in Figure 1.

### 3.2. Results for study 2

The results of the hypothesis testing are presented in Table 1.

#### 3.2.1. Sample characteristics

**Figure 2:**
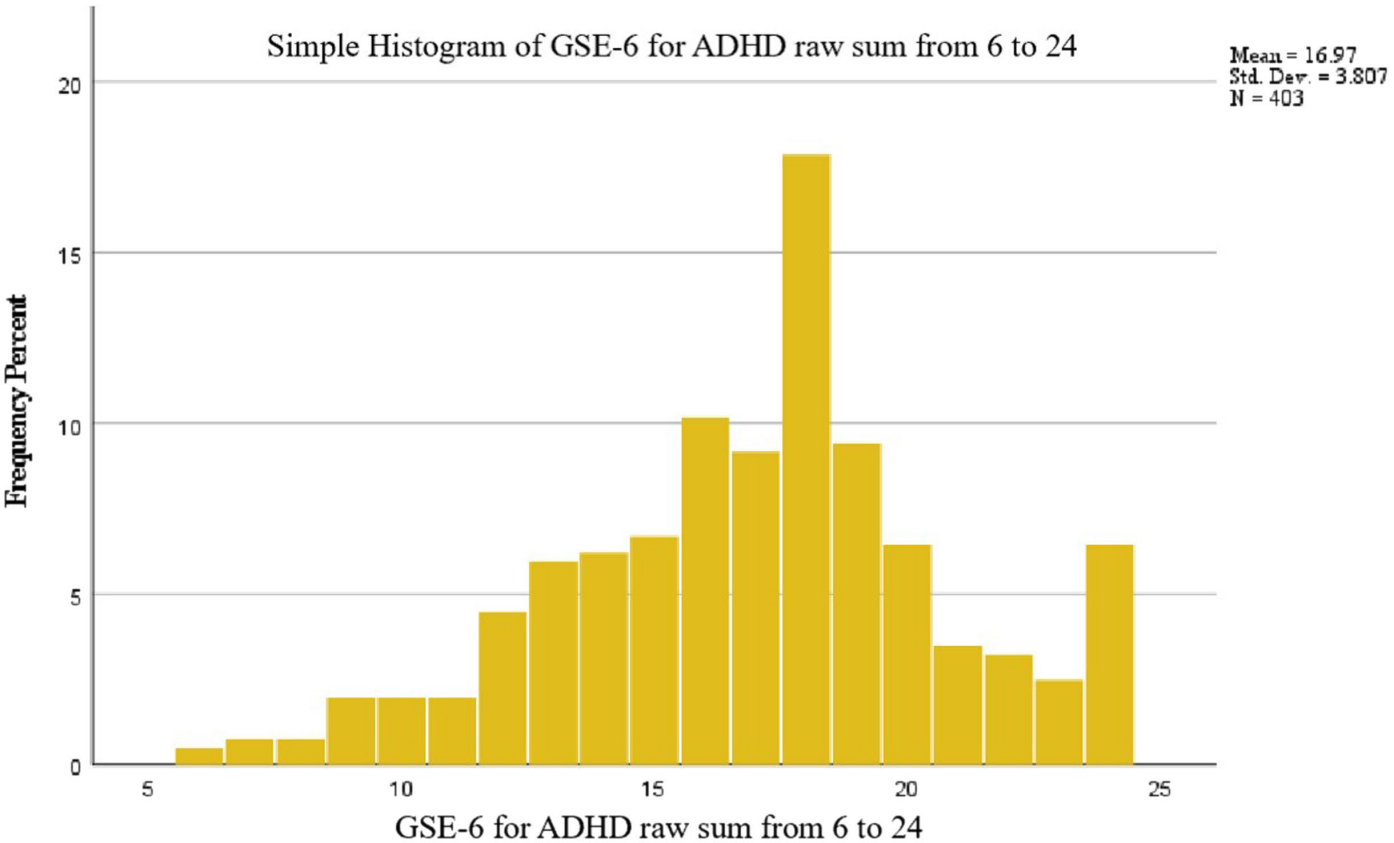
Distribution of the GSE-6 for adults with ADHD total score.

**Figure 3:**
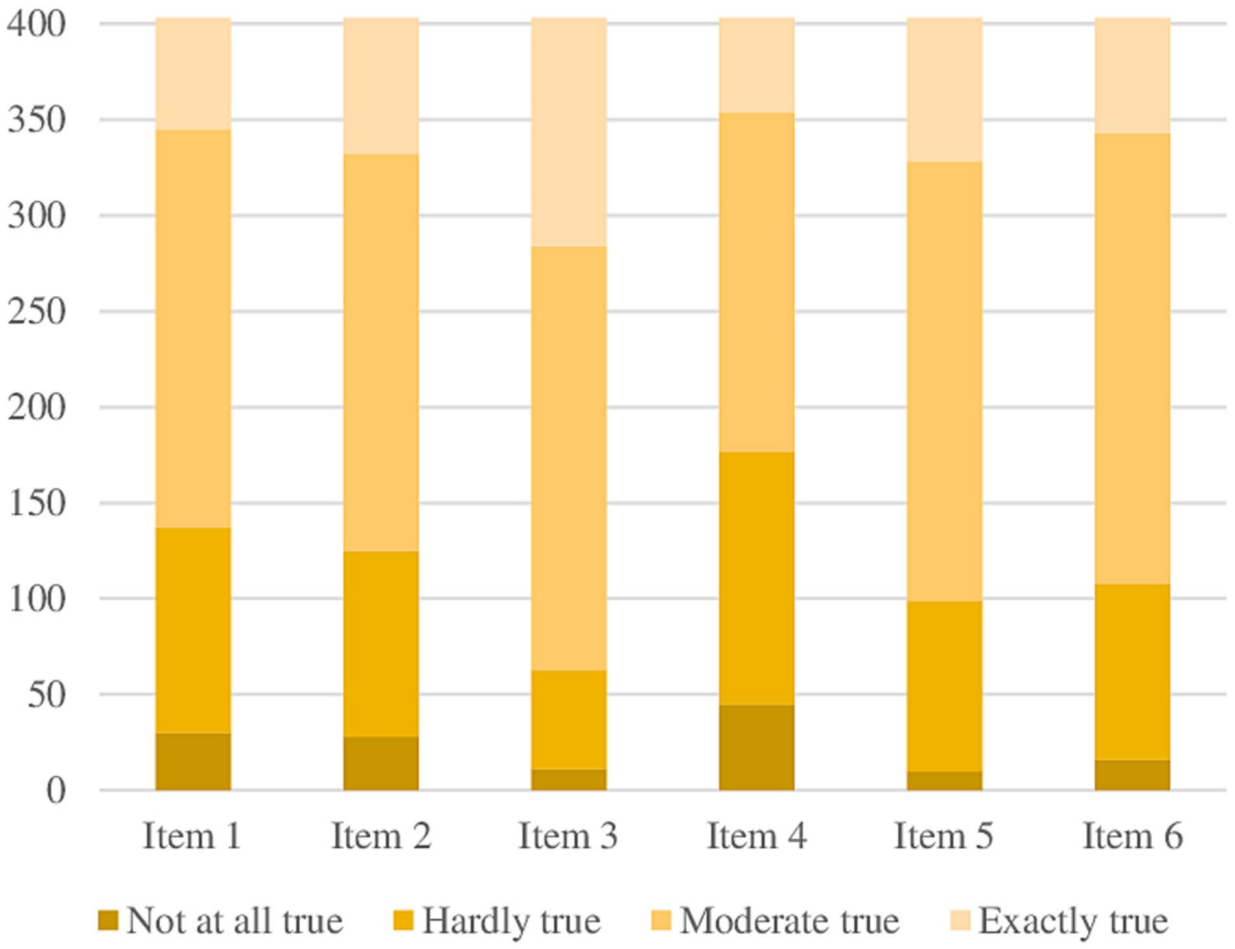
Item distribution for GSE-6 for adults with ADHD.

A total of 403 participants (287 women and 109 men) consented to participate and completed the survey. Table 2 presents the socio-demographic characteristics of the sample. There were no missing responses in the dataset. Descriptive statistics of the GSE-6 items for ADHD are presented in Table 3. The mean raw score of the scale was 16.97 (*SD =* 3.807). The distribution of total score for the GSE-6 for ADHD for the sample was normal and is provided in Figure 2. Item distribution for GSE-6 for ADHD is displayed in Figure 3. No floor or ceiling effects were present in the data. The minimum score was achieved by only two participants (0.5%), and the maximum score was achieved by 26 participants (6.5%).

**Table 2:** Descriptive statistics and socio-demographics of respondents.

Characteristics	Frequency (*n*)	Percentage (*%*)
Gender		
Female	287	71.2
Male	109	27
Do not want to answer	7	1.7
*Total*	403	
Age		
18–24	35	8.7
25–29	42	10.4
30–34	51	12.7
35–39	60	14.9
40–44	80	19.9
45–49	57	14.1
50–54	40	9.9
55–59	21	5.2
60–64	8	2.0
Over 65	9	2.2
*Total*	403	
Marital status		
Not married	109	27
Married/have partner	250	62
Divorced/separated	40	9.9
Widow/widower	4	1
*Total*	403	
Educational level		
Primary/secondary school	159	39.5
High school/ up to 3 years of university	157	39.0
Master’s degree or more	87	21.6
*Total*	403	
Work status		
Student	56	13.9
Paid work	220	54.6
Sick leave	62	15.4
Welfare benefits	10	2.5
Other	55	13.6
*Total*	403	
*Descriptive statistics*	Mean	SD
WHO-5 (0–25)	10.72	5.03
PHQ-4 (0–12)	5.53	3.31

**Table 3:** Characteristics of individual items of the GSE-6 for patients with ADHD.

Item	Mean	*SD*	Factor loading	Correlated Item-total correlation	Cronbach’s α if item deleted
Item 1: “I am confident that I could deal efficiently with unexpected events”	2.73	0.797	0.936	0.805	0.881
Item 2: “Thanks to my resourcefulness, I know how to handle unforeseen situations”	2.80	0.809	0.919	0.797	0.882
Item 3: “I can solve most problems if I invest the necessary effort”	3.11	0.723	0.772	0.668	0.901
Item 4: “I can remain calm when facing difficulties because I can rely on my coping abilities”	2.57	0.845	0.871	0.772	0.886
Item 5: “When I am confronted with a problem, I can usually find several solutions”	2.92	0.707	0.783	0.682	0.866
Item 6: “I can usually handle whatever comes my way”	2.84	0.716	0.846	0.739	0.891

#### 3.2.2. Factor structure

**Figure 4:**
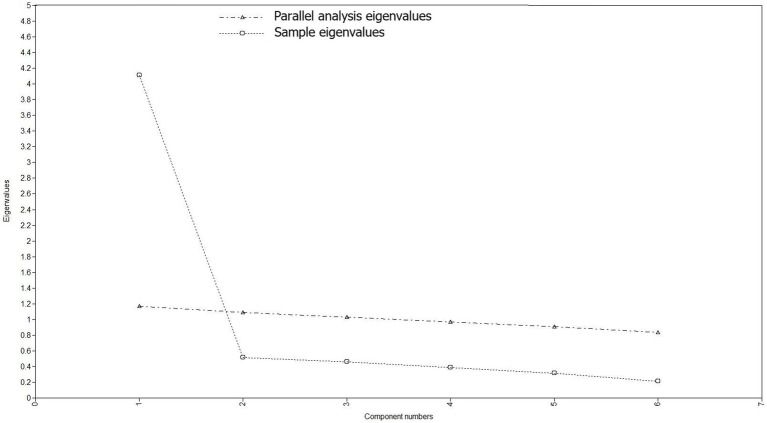
Scree plot of GSE-6 for adults with ADHD.

The EFA supported a one-factor solution by several criteria. One factor had an eigenvalue above 1 (4.624), and inspection of the scree plot and parallel analysis also suggested one factor to be extracted, see Figure 4. The fit indices for a one-factor solution were RMSEA = 0.101 [90% CI 0.073–0.131], CFI = 0.994, TLI = 0.991, SRMR = 0.030. The fit indices were in the acceptable range except for the RMSEA.

The EFA also tested a two-factor solution, which reported item 1 and item 2 to load on factor 1 and the four remaining items to load on factor 2. This model gained following model fit indices: RMSEA = 0.035 [90% CI 0.000–0.089], CFI = 1.000, TLI = 0.999, SRMR = 0.011. However, based on the Kaiser criterion of eigenvalues to exceed 1, inspection of scree plot and parallel analysis, that only two indicators loaded on factor 1, and that the two-factor solution was not considered theoretically meaningful, we decided to retain the one-factor solution.

The one-factor solution obtained in the EFA was tested in a CFA in a sample where participants with unknown gender had been removed, to further allow for test of measurement invariance across men and women. The CFA showed acceptable fit indices: RMSEA = 0.097 [0.069–0.127], CFI = 0.995, TLI = 0.992, SRMR = 0.018.

In test of measurement invariance, the model was first fitted separate to women and men (Table 4). Fit indices indicated good model fit, except for the RMSEA for men which was above 0.10. However, as the RMSEA has been demonstrated to indicate worse fit in models with small df and low sample size (56), and because the CFI, TLI and SRMR values were in the acceptable range, we proceeded to test for measurement invariance in the complete sample. Configural invariance was achieved as the one-factor structure had adequate model fit in both samples. The metric model with factor loadings constrained equal across women and men did not show deterioration in fit indices and was retained. In the final step factor loadings and item thresholds were constrained equal across women and men to test scalar invariance. This latter model showed a slight deterioration in the fit indices, but this change was considered marginal, and the model was retained.

**Table 4:** Test of measurement invariance (*n_women_* = 287; *n_men_* = 109).

Model	Test	Compared with	*χ^2^*(df)	RMSEA	CFI	TLI	SRMR	Δ*χ^2^*(df)	*p*	ΔCFI	ΔRMSEA	ΔSRMR
M1a	Women		25.824 (9)	0.081 [0.045, 0.118]	0.997	0.995	0.019					
M1b	Men		21.629 (9)	0.113 [0.052, 0.176]	0.993	0.989	0.025					
M2	Configural		47.485 (18)	0.091 [0.060, 0.123]	0.996	0.993	0.021					
M3	Metric	M2	39.679 (23)	0.061 [0.026, 0.092]	0.998	0.997	0.023	3.900 (5)	0.564	0.002	−0.030	−0.002
M4	Scalar	M3	64.260 (34)	0.067 [0.041, 0.092]	0.996	0.996	0.024	19.280 (11)	0.056	−0.002	0.006	0.001

#### 3.2.3. Internal consistency

The GSE-6 for ADHD demonstrated good internal consistency among adults in a Norwegian sample (Cronbach’s α coefficient equal to 0.907 [95% CI 0.892–0.920]) (57). In the inter-item correlation matrix, the corrected item-total correlation ranged from 0.668 to 0.805. Cronbach’s α coefficient if an item was deleted was counted for each item and ranged from 0.881 to 0.901. More information is provided in Table 4.

#### 3.2.4. Correlation between scales

As hypothesized, we found a moderate positive correlation of the GSE-6 and WHO-5 (*r_s_* = 0.578, *p* < 0.001) and a moderate negative correlation between the GSE-6 and PHQ-4 (*r_s_* = −0.595, *p* < 0.001). Correlation coefficients are presented in Table 1. The mean and *SD* of the WHO-5 and PHQ-4 are listed in Table 2.

## 4. Discussion

The purpose of this study was to develop and validate a condensed version of the GSE scale to assess the overall self-efficacy in adults with ADHD. We developed a six-item version of the GSE-10 by reducing it and retaining the six items deemed most relevant for adults with ADHD by an expert panel including individuals with ADHD and mental health professionals.

Face validity was assessed using QQ-10 questionnaire. The value domain received is comparable to other studies that used QQ-10 to validate measurement scales (36, 58–61). Item 3 obtained the highest mean in the value domain of the QQ-10, indicating that the testers found the new measuring tool easy to use. The burden domain also received a value comparable to other studies (35, 36, 58, 61, 62). Only 16 raters completed the QQ-10, which is a relatively small group of evaluators. However, the QQ-10 evaluation of the GSE-6 for ADHD indicates promising results, as the scale was easy-to-administer and user-friendly, revealing that the participants had a pleasant experience using the GSE-6 for ADHD.

We evaluated the reliability of the GSE-6 for ADHD, finding good quality data without missing values. The questionnaire demonstrated good internal consistency, which is in line with previous studies of the GSE-10, with Cronbach’s α values ranging from 0.78 to 0.95 (22, 24, 40, 41, 63–67). Correlated item-total correlation in various studies ranged from 0.25 to 0.63 (41) and 0.36 to 0.52 (22) to 0.63 to 0.73 (67). Our scale displayed higher values of this parameter, from 0.668 to 0.805. In addition, the results did not find an increase in Cronbach’s α if any of the items were removed, consistent with the results for the GSE-10 scale reported by Dahlberg et al. (65). This finding demonstrates that the reduction from 10 to six items did not deteriorate the internal consistency, and that the six retained items form a reliable scale.

Using EFA and CFA, a one-factor solution was favored based on several criteria. Most previous studies evaluating the factor structure of the GSE-10 have supported a one-factor structure (22–24, 40, 41, 63–67). The results of this study align with these previous findings, and it is promising that the brief GSE-6 for ADHD has the same factor structure as the 10-item scale. The eigenvalue corresponds to other studies of the GSE-10, which have ranged from 4.9 (68) to 6.96 (39, 40). The EFA factor loadings of the indicators in this study ranged from 0.772 to 0.936, which is equal to or somewhat higher than in previous studies (24, 41, 64). Test of measurement invariance indicated that configural, metric and scalar invariance was supported across men and women. However, as the low sample size did not allow for splitting the data, the EFA, CFA and test of measurement invariance were conducted on the same sample. This is discouraged due to risk of overfitting or inflated model fit indices (69). Moreover, although most fit indices indicated satisfactory model fit for the CFA and test of measurement invariance, the RMSEA exceeded recommended thresholds. The results should, therefore, be considered tentative and must be replicated in studies with larger samples.

The construct validity of the GSE-6 for ADHD was assessed by investigating the correlation with other relevant measures. A negative moderate correlation between the GSE-6 for ADHD and depressive affect/anxiety measured with the PHQ-4 corresponds to previous studies. Luszczynska et al. (42) conducted a validation study of GSE-10 on 1933 participants from Poland, Germany and South Korea, and found a negative correlation of GSE-10 with negative affect. Nilsson et al. (40) also found a negative correlation between GSE-10 and depressive symptoms (*r* = −0.42). Another Norwegian study in line with our results was conducted by Leganger et al. (41). In this study, a negative correlation was also found between the GSE-10 scale and negative affect (*r* = −0.21) (41). The negative correlation between GSE-6 and PHQ-4 is also in line with those reported by Romppel et al. (31). The assessment of depression symptoms in their study was conducted using the PHQ-9 scale, where the correlation with GSE was −0.35. The study also used the Hospital Anxiety and Depression Scale (70), where the correlation was −0.35 with the anxiety domain, and − 0.45 with the depression domain. The correlation between the GSE-6 for ADHD and PHQ-4 in this study is stronger than those obtained by Romppel et al. (31). However, in this study we used the PHQ-4, which measures anxiety and depression combined; thus, the difference in correlation coefficients may be because we did not assess anxiety and depression separately.

A positive moderate correlation between general self-efficacy and well-being measured with the WHO-5 is consistent with previous studies (41, 42, 63). However, our results exhibited a stronger positive correlation of general self-efficacy and well-being compared to previous studies. This outcome can be explained both by the variety of scales used to measure well-being, and by the fact that an adult ADHD population may have unique characteristics compared to other studied populations. In general, the results support previous research that has found a positive relationship between general self-efficacy and well-being (41, 42, 63). This relationship is particularly pronounced in patients with ADHD, confirming the need to pay more attention and resources on the development of self-efficacy in this group of patients.

Romppel et al. (31) also validated a six-item version of the GSE-10 in a sample of patients at risk for heart failure. In their study, they kept GSE-10 Items 2, 3, 4, 5, 7, and 10. The decision on what items to select was different than in the present study. Romppel et al. (31) selected six items based on the highest coefficient of variation and good discrimination of participants at different levels of self-efficacy. Bonsaksen et al. (32) developed the GSE-7 (general self-efficacy scale consisting of 7 items), which uses items 4 to 10 from GSE-10. The scale was developed for the Norwegian adult population with morbid obesity, and the Rasch model was used to select the items (32). Their scale also displayed a unidimensional structure, explaining 64.5% variance. In the present study, based on the consensus reached in an expert panel of adults with ADHD and mental health professionals, we kept the GSE-10 items number 4, 5, 6, 7, 8, and 10. The two GSE-6 scales for adults with ADHD and those at risk for heart failure were constructed with different items from the GSE-10, complicating the direct comparison between these two scales. The GSE-6 for ADHD and GSE-7 differ in only one item, Item 9; however, we believe that, for patients with ADHD, a decrease in one item can play a significant role in the perception of the face validity of the scale.

Our findings suggest that the GSE-6 is an easy-to-administer, acceptable, concise, valid, and reliable self-rated tool for measuring self-efficacy among adults with ADHD. As such, the GSE-6 is recommended for use in clinical settings as an assessment tool, aiding mental healthcare professionals, therapists and clinicians in understanding the patient’s self-efficacy in an understudied ADHD adult population. Furthermore, the identified correlations between self-efficacy, well-being and mental health contribute to a more comprehensive understanding of the role of self-efficacy in a clinical setting. These findings can guide clinical practice and future research by contributing to the development of educational interventions and treatment approaches fostering self-efficacy and psychological well-being.

### 4.1. Strengths and limitations of the study

A particular strength of the current study was the participation of patient representatives in adapting the GSE-6 for ADHD. This ensures that the patient perspective related to language perception, accessibility of understanding and relevance of the scale items is preserved. The use of the QQ-10 to evaluate the content validity of the GSE-6 is a strength of the study, nevertheless, for a more accurate assessment of face validity, qualitative interviews should be conducted. In addition, only 16 people completed the QQ-10 questionnaire, which is a limitation. However, even though the sample might not entirely represent the ADHD-population, these participants were patient collaborators and user representatives from a wide geographical area in Mid Norway. Yet, this sample’s size limitation should be considered when designing future GSE-6 validation studies. Further studies with a larger number of participants are required to assess the convenience and ease of use of the GSE-6 for ADHD.

In the second phase, participants reported to have been diagnosed with ADHD. However, the ADHD-diagnosis was not confirmed through structured clinical interviews. Therefore, further investigation on the content and construct validity of GSE-6 in a clinical sample of adults with confirmed ADHD is recommended. Due to the cross-sectional design, we could not evaluate the test–retest reliability of the GSE-6 for ADHD, which is a critical psychometric component, and future studies should use a prospective design to evaluate this. Even though we have a sufficiently large sample size for conducting EFA (50, 51), the limited sample size did not allow for the test of EFA and CFA in separate samples. Therefore, the factor structure identified in the current study should be replicated in future studies (ideally with larger samples).

## 5. Conclusion

This study reports the development and assessment of the validity of the GSE-6 for ADHD, which aims to measure the general self-efficacy of those with ADHD. The 6-item GSE was developed in collaboration with user representatives from the Norwegian user-led ADHD organization, and its content validity was assessed in a nonclinical adult ADHD population using the QQ-10 questionnaire, demonstrating a high positive value score and low negative burden score. The GSE-6 for ADHD demonstrated one-factor structure, and moderate correlations with measures of well-being and symptoms of depression and anxiety were found. Taken together, the results support using the GSE-6 for ADHD to measure general self-efficacy in an adult ADHD population. Future studies should evaluate the scale in clinical populations.

## Data availability statement

The dataset analysed during the current study can be made available from the corresponding author on reasonable written request.

## Ethics statement

The studies involving humans were approved by the Regional Committee for Medical and Health Research Ethics in Central Norway (2021/2081643). The studies were conducted in accordance with the local legislation and institutional requirements. The participants provided their written informed consent to participate in this study.

## Author contributions

TS: writing – original draft. TS, AH, and HH: conceptualization. HH: data curation. TS and AH: statistical analyses. TS, HP, HH, JV, MLL-C, and AH: writing – review and editing. MLL-C: investigation. HH and MLL-C contributed to conceptualization and data collection. MLL-C and AH: supervision. All authors have read and agreed to the published version of the manuscript.
